# Exploration of the shared pathways and common biomarker in adamantinomatous craniopharyngioma and type 2 diabetes using integrated bioinformatics analysis

**DOI:** 10.1371/journal.pone.0304404

**Published:** 2024-06-07

**Authors:** Yibo Han, Yong Wang, Shuo Li, Kohji Sato, Satoru Yamagishi

**Affiliations:** 1 Department of Organ and Tissue Anatomy, Hamamatsu University School of Medicine, Hamamatsu, Japan; 2 Neurosurgery, The First Hospital of China Medical University, Shenyang, China; 3 Department of Optical Neuroanatomy, Institute of Photonics Medicine, Hamamatsu University School of Medicine, Hamamatsu, Japan; The First Affiliated Hospital of Nanjing Medical University, CHINA

## Abstract

Craniopharyngiomas are rare tumors of the central nervous system that typically present with symptoms such as headache and visual impairment, and those reflecting endocrine abnormalities, which seriously affect the quality of life of patients. Patients with craniopharyngiomas are at higher cardiometabolic risk, defined as conditions favoring the development of type 2 diabetes and cardiovascular disease. However, the underlying common pathogenic mechanisms of craniopharyngiomas and type 2 diabetes are not clear. Especially due to the difficulty of conducting in vitro or in vivo experiments on craniopharyngioma, we thought the common pathway analysis between craniopharyngioma and type 2 diabetes based on bioinformatics is a powerful and feasible method. In the present study, using public datasets (GSE94349, GSE68015, GSE38642 and GSE41762) obtained from the GEO database, the gene expression associated with adamantinomatous craniopharyngioma, a subtype of craniopharyngioma, and type 2 diabetes were analyzed using a bioinformatic approach. We found 11 hub genes using a protein–protein interaction network analysis. Of these, seven (DKK1, MMP12, KRT14, PLAU, WNT5B, IKBKB, and FGF19) were also identified by least absolute shrinkage and selection operator analysis. Finally, single-gene validation and receptor operating characteristic analysis revealed that four of these genes (MMP12, PLAU, KRT14, and DKK1) may be involved in the common pathogenetic mechanism of adamantinomatous craniopharyngioma and type 2 diabetes. In addition, we have characterized the differences in immune cell infiltration that characterize these two diseases, providing a reference for further research.

## Introduction

Craniopharyngioma (CP) is a rare central nervous system neoplasm that originates from residual epithelial cells of Rathke’s capsule, which are considered to be histologically benign (WHO grade I) [1]. Histologically, CP exists in two distinct subtypes: adamantinomatous craniopharyngioma (ACP) and papillary craniopharyngioma (PCP) [2]. Unlike PCP, which usually occurs only in adults, patients with ACP usually present with symptoms in one of two age ranges, 5–15 and 45–60 years of age, and ACP thus has a distinct bimodal distribution [3–5]. Although the incidence of CP is very low, with 0.5–2.5 cases per million people, childhood-onset CP accounts for 1.2–4.6% of all intracranial tumors in pediatric patients [2, 3, 6]. CPs typically develop along the pituitary-hypothalamic axis, between the sella turcica and the third ventricle [7]. Owing to the relationship between the CP and these important brain structures, and in particular the hypothalamus, patients often show a variety of symptoms, such as headache, visual impairment, and those relating to endocrine abnormalities, both before and after surgery, and these severely affect their quality of life [8, 9].

Type 2 diabetes (T2D) is a metabolic disorder characterized by relative insulin deficiency owing to a combination of impaired pancreatic beta-cell function and insulin resistance in target organs. This form is responsible for >90% of all cases of diabetes [10]. Patients with CP are at higher cardiometabolic risk: there is a 3–19-fold higher incidence of cardiovascular mortality associated with the metabolic syndrome in patients with CP than in the general population. Cardiometabolic risk is defined as conditions favoring the development of T2D and cardiovascular disease, which is the most frequent cause of morbidity and mortality associated with T2D [11–14]. Patients with CP have a greater disease burden associated with T2D, cerebral infarction, fracture, and serious infection. A recent population-based study conducted in Sweden revealed that patients with CP have 7-, 5-, and 6-times higher incidences of cerebral infarction, cerebrovascular disease mortality, and T2D, respectively [6]. However, the common features and molecular mechanisms of the development of ACP and T2D have not been elucidated.

In recent years, advancements in sequencing technology and bioinformatic analytical techniques have enabled us to explore the shared pathogenic mechanisms of various diseases at the genetic level. In the present study, we analyzed datasets from patients with ACP or T2D that were obtained from the Gene Expression Omnibus (GEO) database. Integrated bioinformatic approaches were used to identify common differentially expressed genes (DEGs), and functional enrichment analysis was performed to investigate their roles in both ACP and T2D. We utilized weighted gene co-expression network analysis (WGCNA) to identify co-expression modules and genes with similar expression profiles in ACP and T2D. Furthermore, we constructed protein–protein interaction (PPI) networks using STRING and Cytoscape, analyzed gene modules, and thereby identified 11 hub genes. We then screened the hub genes by least absolute shrinkage and selection operator (LASSO) analysis, a more concise and generalized gene set is obtained, called the model genes. Furthermore, we compared the differences in expression, and ultimately identified genes that may be involved in the shared expression that characterizes ACP and T2D, by means of single-gene validation and Receiver operating characteristic (ROC) analysis. Finally, we performed immune cell infiltration analysis on the DEGs and analyzed the relationships between hub genes in 28 immune cell types in order to better understand the abnormalities in immune cells that characterize these two diseases.

## Materials and methods

### Dataset

The GEO (www.ncbi.nlm.nih.gov/geo) is a large, free, and publicly accessible database that contains gene expression data for various diseases [15]. The GSE94349 dataset includes 168 gene expression datasets, including information regarding 24 ACP tumors and 27 non-tumor tissue samples [16], and the GSE68015 dataset comprises 112 gene expression datasets, including information regarding 15 ACP tumors and 16 non-tumor tissue samples [17]. Both of these datasets were obtained using Affymetrix HG-U133plus2.0 chips (Platform GPL570; Santa Clara, CA, USA). GSE38642 contains gene expression data for 54 normal samples and nine samples from patients with T2D [18], and GSE41762 contains gene expression data for 57 samples from normal patients and 20 samples from patients with T2D [19]. The samples comprising these two datasets were obtained from human pancreatic islets, and the gene expression data were generated using the Affymetrix Human Gene 1.0 ST Array (Platform GPL6244).

### WGCNA

WGCNA is an algorithm that is used for the analysis of gene expression patterns in large datasets. It clusters genes on the basis of similar expression patterns, constructs gene modules, and analyzes the associations between expression modules and biological traits using a weighted approach [20]. In the present study, we removed outlier samples from the hierarchical clustering analysis using the Hclust function, and then WGCNA analysis was performed on the GSE94349 and GSE38642 datasets using the package in R (R Foundation for Statistical Computing, Vienna, Austria) to obtain expression modules associated with ACP and T2D. An appropriate soft-thresholding power, β = 7, was calculated using R^2^>0.90 to achieve scale-free topology. Next, co-expressed modules were identified using hierarchical clustering, and a hierarchical clustering tree was obtained. The minimum module size was set to 100, and modules with correlations >50% were selected. Finally, module eigengenes were identified and the relationships between module eigengenes and clinical features were evaluated, yielding expression profiles for the characteristic genes of each module. We focused on highly correlated modules and selected genes from these modules for further analysis. Pearson correlation coefficients and *p*-values were calculated to determine the key modules associated with ACP on the basis of the relationships between module eigengenes and disease features.

### LASSO and ROC

LASSO is a statistical method that aims to elucidate specific relationships between two related variables that has a dimensionality reduction effect when compared with conventional analyses. Performing LASSO analysis on genes identified using WGCNA improves the accuracy with which genes associated with target features are selected [21]. In our research, we used the function in the "glmnet" package to fit the training data and obtained the LASSO model. During the fitting process, we set "maxit = 10000" to ensure that the model can fully converge. Then, we use the "cv.glmnet()" function to perform cross-validation and select the optimal regularization parameter (lambda) by setting "lambda = 1000" and "alpha = 1".Finally, model genes were obtained, and were used for further analysis and validation. And, we used the “ROCR” package to draw the ROC (Receiver Operating Characteristic) curve to evaluate the performance of our trained LASSO model. Finally, we used the “plot()” function to visualize the ROC curve.

### Acquisition and analysis of DEGs

DEGs were identified using the “limma” R package, with Log |FC| > 1 and *p* < 0.05 being considered to indicate statistical significance. DEGs were identified from four datasets, and intersections of the DEGs among these datasets were identified after removing non-intersecting genes. The resulting dataset was used for further analysis.

### Functional enrichment analysis

The “clusterProfiler” and “enrichplot” packages in R were used to conduct Gene Ontology (GO) analysis, Kyoto Encyclopedia of Genes and Genomes (KEGG) analysis, and Gene Set Enrichment Analysis (GSEA) on the DEGs [22]. Results with *p* < 0.05 were considered to be statistically significant, and the enriched terms were visualized using appropriate plots or diagrams. The “org.Hs.eg.db” package was used for gene annotation [23].

### Protein–protein interaction network construction and hub gene identification

STRING (version 11.5) is an online tool for the identification of PPIs and the construction of a PPI network. We set a threshold for the interaction score of > 0.4 [24] and Cytoscape software (version 3.9.0; Cytoscape Consortium, San Diego, CA, USA) was used to visualize the PPI network [25]. We used the MCODE plugin in Cytoscape to analyze the PPI network and identify core functional modules and hub genes. The parameter settings were as follows: degree cut-off = 2, max depth = 100, node score cut-off = 0.2, and K-core = 2 [26].

### Analysis of immune cell infiltration

We quantified the relative infiltration levels of 28 immune cell types in the GSE94349 and GSE38642 datasets using the ssGSEA algorithm [27]. The immune gene sets from the Molecular Signatures Database (MsigDB) were used as references for ssGSEA, and a significance threshold of *p* < 0.05 and a false discovery rate (FDR) q-value of < 0.05 were used for the enrichment analysis. Box plots were used to display the differential expression levels of 28 types of infiltrating immune cells. Spearman correlation coefficients were calculated for the relationships between the 28 types of cells and hub genes, and we performed visualization using the “ggplot2” package.

### All datasets are obtained from public databases: GEO database

GSE94349:

https://www.ncbi.nlm.nih.gov/geo/query/acc.cgi?acc=GSE94349

GSE68015:

https://www.ncbi.nlm.nih.gov/geo/query/acc.cgi?acc=GSE68015

GSE38642:

https://www.ncbi.nlm.nih.gov/geo/query/acc.cgi?acc=GSE38642

GSE41762:

https://www.ncbi.nlm.nih.gov/geo/query/acc.cgi?acc=GSE41762

## Results

### Identification of DEGs in patients with ACP or T2D

**Fig 1:**
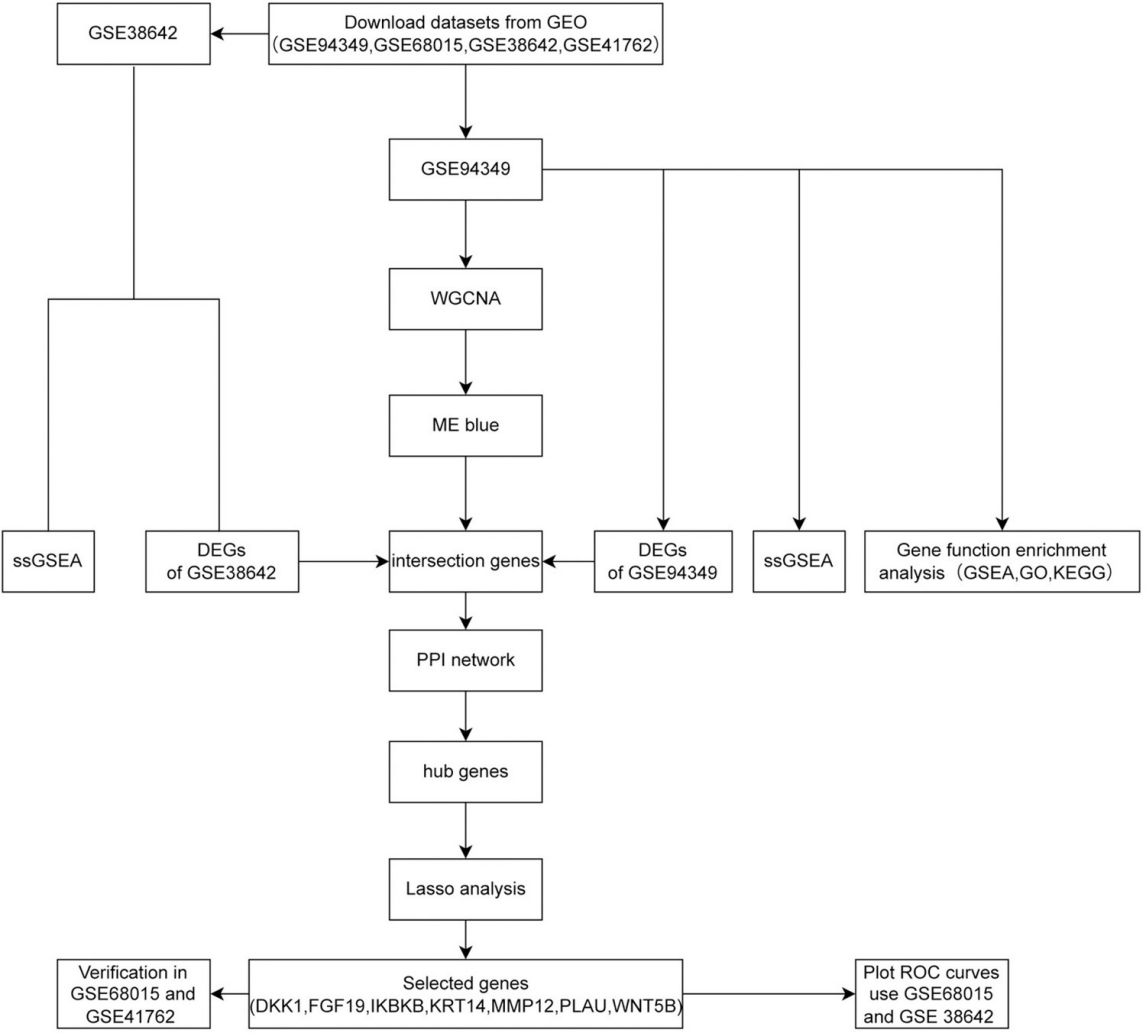
Flow chart of the research design.

**Fig 2:**
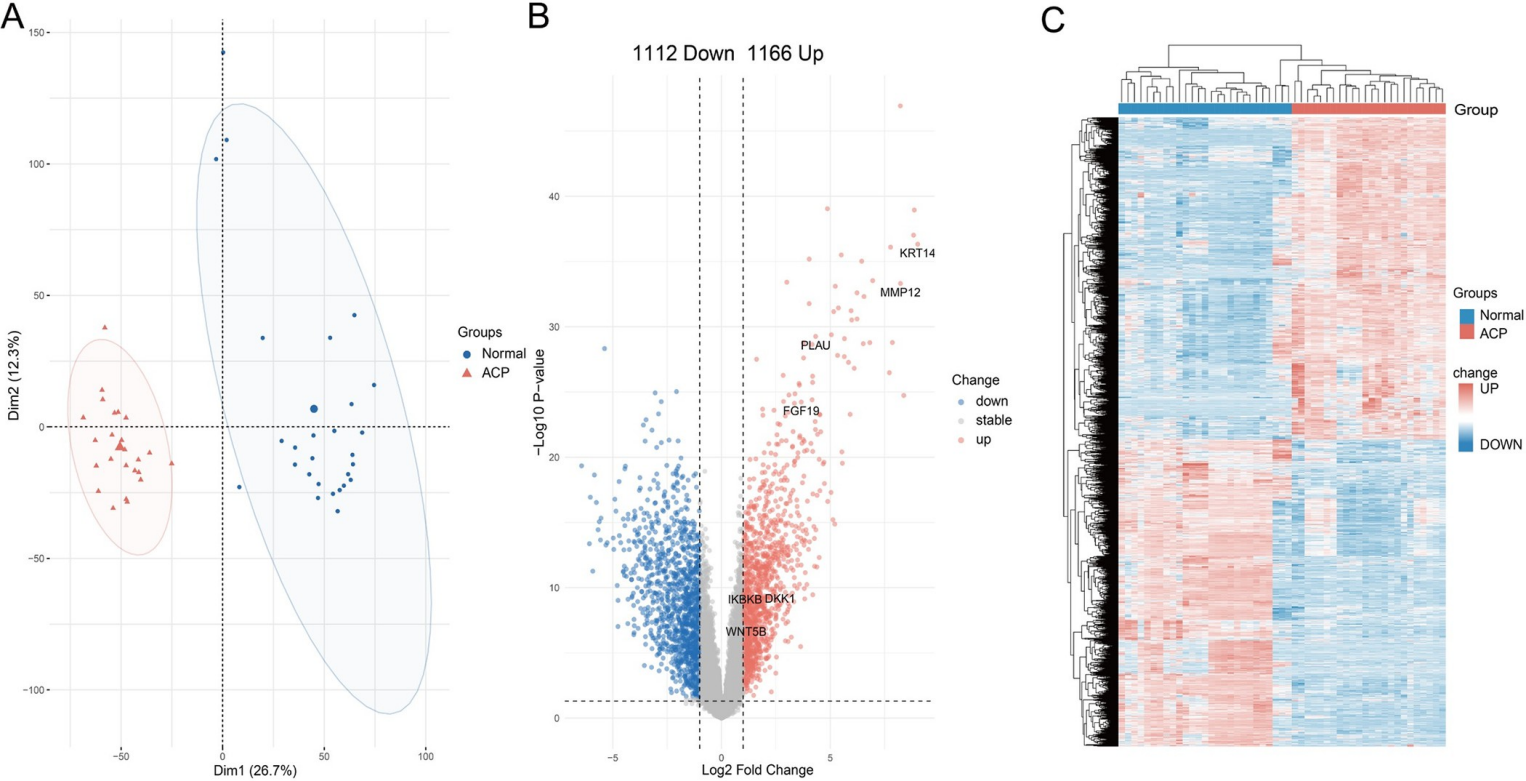
Evaluation of the GSE94349 dataset and acquisition of differentially expressed genes. (A) Results of the principal components analysis. (B) Volcano plot of the differentially expressed genes. (C) Heatmap of the differentially expressed genes.

We provide a flow chart of the research design (Fig 1). Principal components analysis revealed that the expression data in the ACP dataset featured close associations and a high level of reliability (Fig 2A). To identify DEGs between the ACP and control groups, we conducted differential expression analysis using the “limma” package. Analysis of the GSE94349 dataset identified 1,166 upregulated genes and 1,112 downregulated genes (Fig 2B), and the resulting heatmap shows distinct expression patterns of ACP tumor tissues and non-tumor tissues (Fig 2C).

### Results of WGCNA

**Fig 3:**
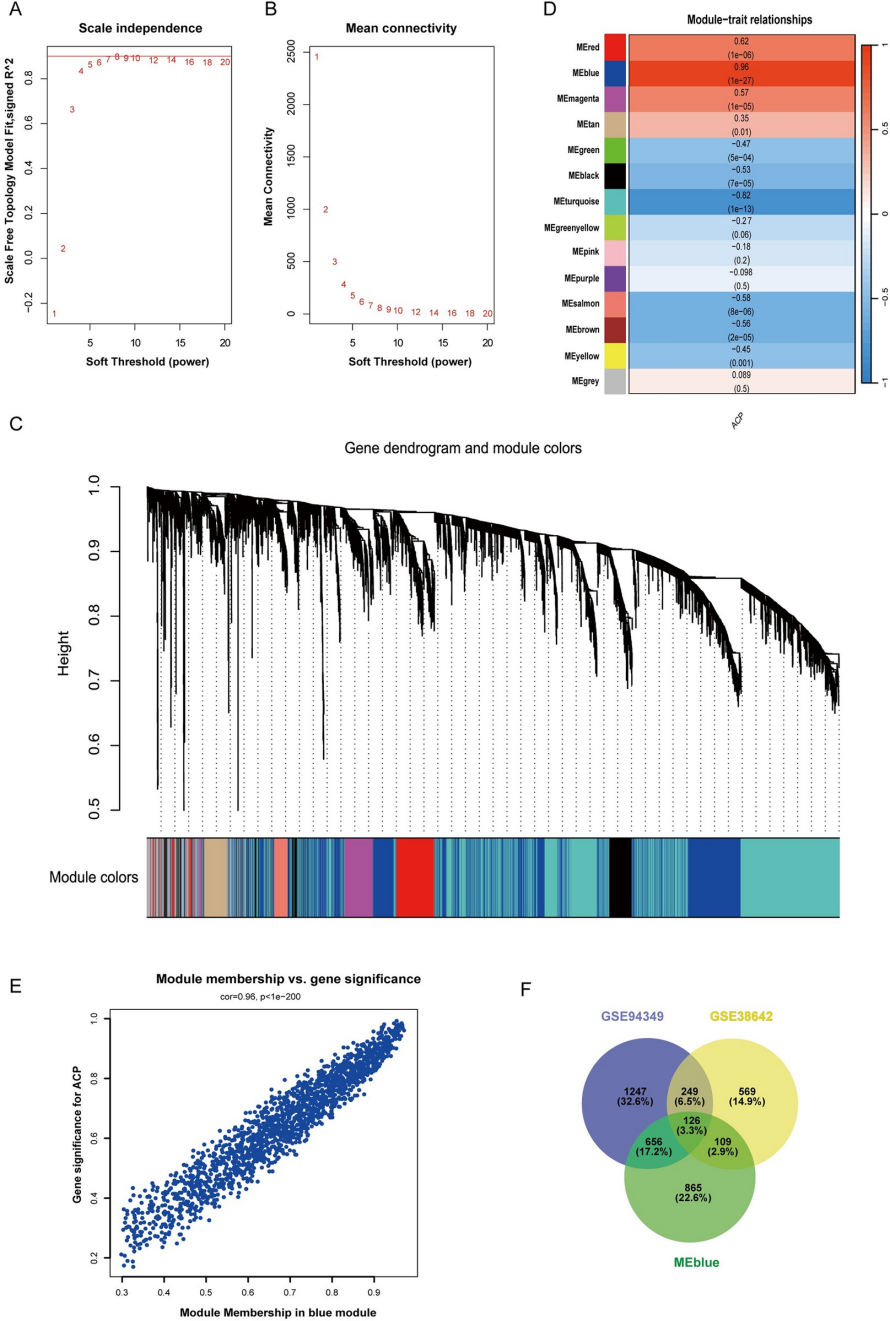
Results of the weighted gene co-expression network analysis of gene modules. (A–B) Scale-free fit indexes and mean connectivities for the various soft-thresholding powers. (C) Module–trait relationships. The meaning of the numbers in each grid refers to the correlation coefficient and p-value of the corresponding module. (D) Clustering dendrogram for the genes. Each branch represents a gene, and each color represents a co-expression module. (E) Scatter plot for the correlations between gene module membership in the blue module and significance level. (F) Venn diagram showing the member genes of the blue module and the differentially expressed genes from the GSE94349 and GSE38642 datasets.

To further evaluate the genes associated with ACP, we performed an analysis using the “WGCNA” package in R on the GSE94349 dataset to identify ACP-related modules. We selected the top 10,000 genes on the basis of the largest median absolute deviation values. Prior to the analysis, we used the “Hclust” function to remove outlier samples from the hierarchical clustering analysis. Based on the criterion of R^2^ > 0.9, an appropriate soft-thresholding power β (β = 7) was selected to achieve scale-free topology (Fig 3A and 3B), and a hierarchical clustering tree was used to identify co-expression modules (Fig 3C). The minimum module size was set to 100, and modules were defined using a correlation threshold of >50%. Finally, module eigengenes were identified and their relationships with clinical features were calculated to obtain the expression profile for each module. Hierarchical sample clustering was performed with a height cut-off of 180 and a minimum cluster size of 10 samples. A total of 13 modules were identified (Fig 3D). Among these, the MEblue module (r = 0.96, *p* = 1e^−27^) showed a positive relationship with ACP (Fig 3E), and 1,756 genes in the Blue module were used for further analysis. DEGs were independently obtained from the GSE94349 and GSE38642 datasets, and the intersection of DEGs with the genes in the Blue module was visualized using a Venn diagram (Fig 3F). This yielded 126 overlapping genes that were considered to be key genes associated with ACP.

### PPI networks

**Fig 4:**
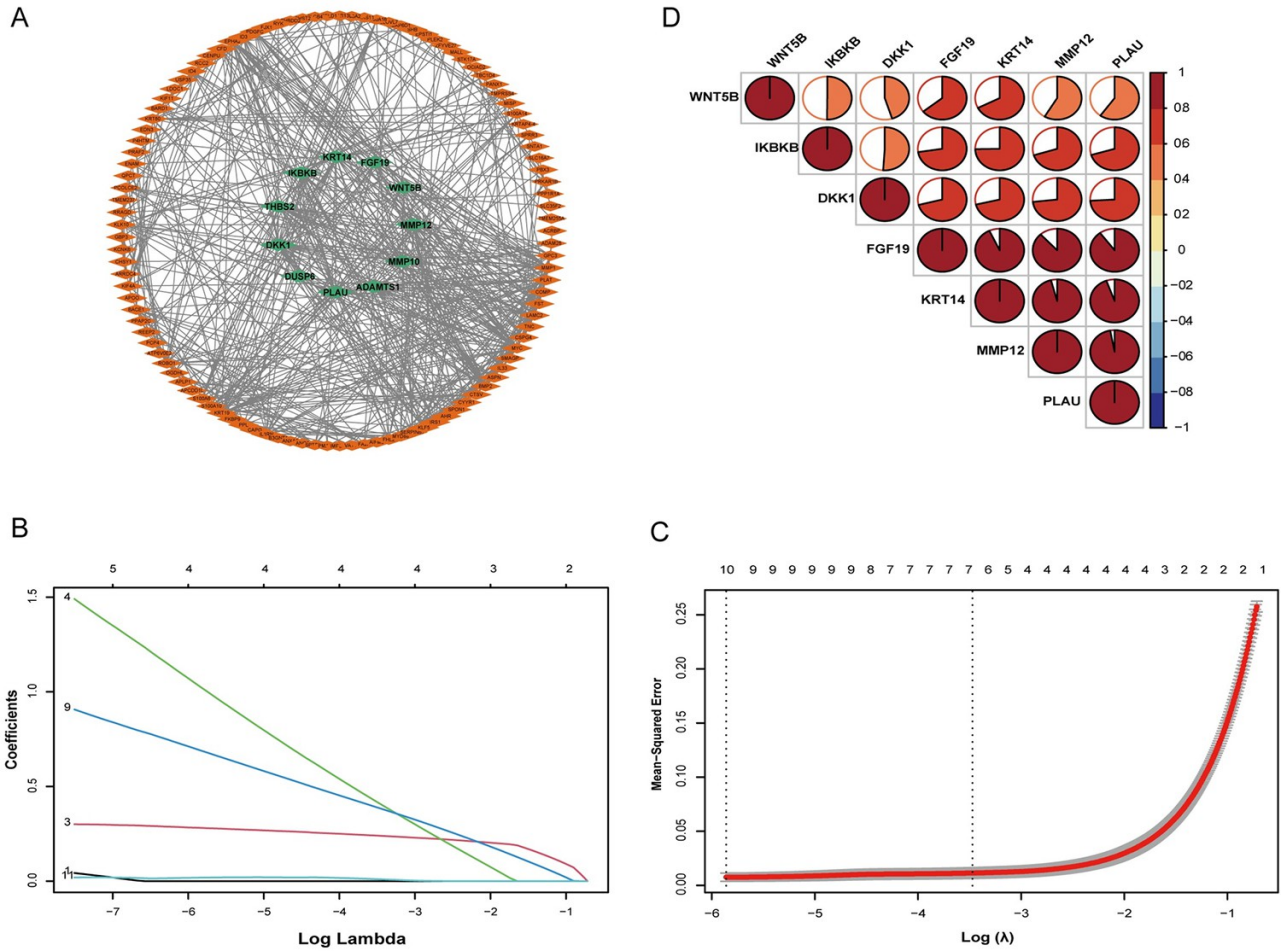
Identification of model genes. (A) Protein–protein interaction network. Quadrilaterals represent proteins and lines represent interactions between proteins. The 11 green quadrilaterals in the center represent the hub genes. (B) Coefficient path diagram. (C) Cross validation score. Dotted vertical lines were drawn at the optimal values using the minimum criteria (lambda.min, left) and the one standard error of the minimum criterion (1-SE criterion, right). (D) Correlations among the model genes. The depth of the color and the area occupied by the circle are used to describe the correlation.

PPI network analysis of the intersecting genes was performed using STRING, and the resulting PPI network was further analyzed using Cytoscape. Functional modules related to the network were identified using the MOCDE tool. A functional module composed of 11 genes (*DKK1*, *MMP12*, *KRT14*, *PLAU*, *WNT5B*, *IKBKB*, *FGF19*, *THBS2*, *MMP10*, *DUSP6*, and *ADAMTS1*) as hub genes was identified and further analyzed (Fig 4A).

### Analysis and validation of the hub genes

**Fig 5:**
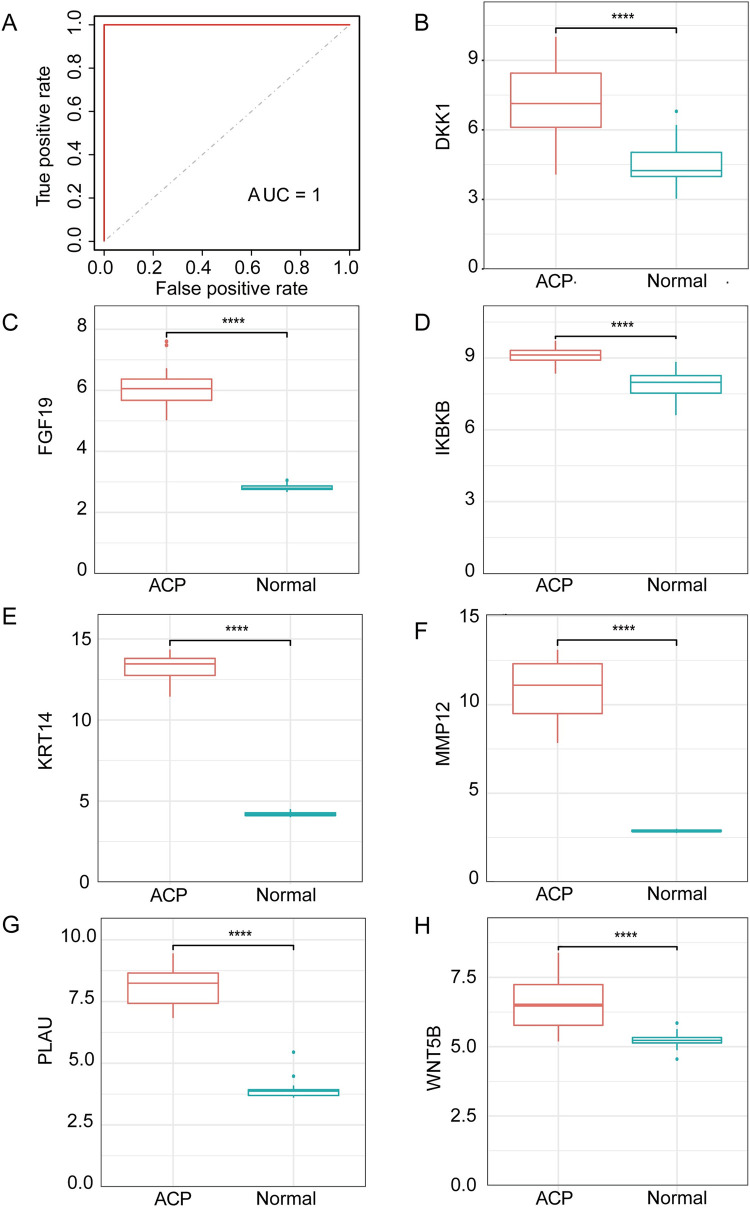
Validation of the model genes for adamantinomatous craniopharyngioma (ACP). (A) Receiver operating characteristic curves for ACP-related model genes from the GSE68015 dataset. (B–H) Boxplots of model gene expression in the dataset GSE68015.

**Fig 6:**
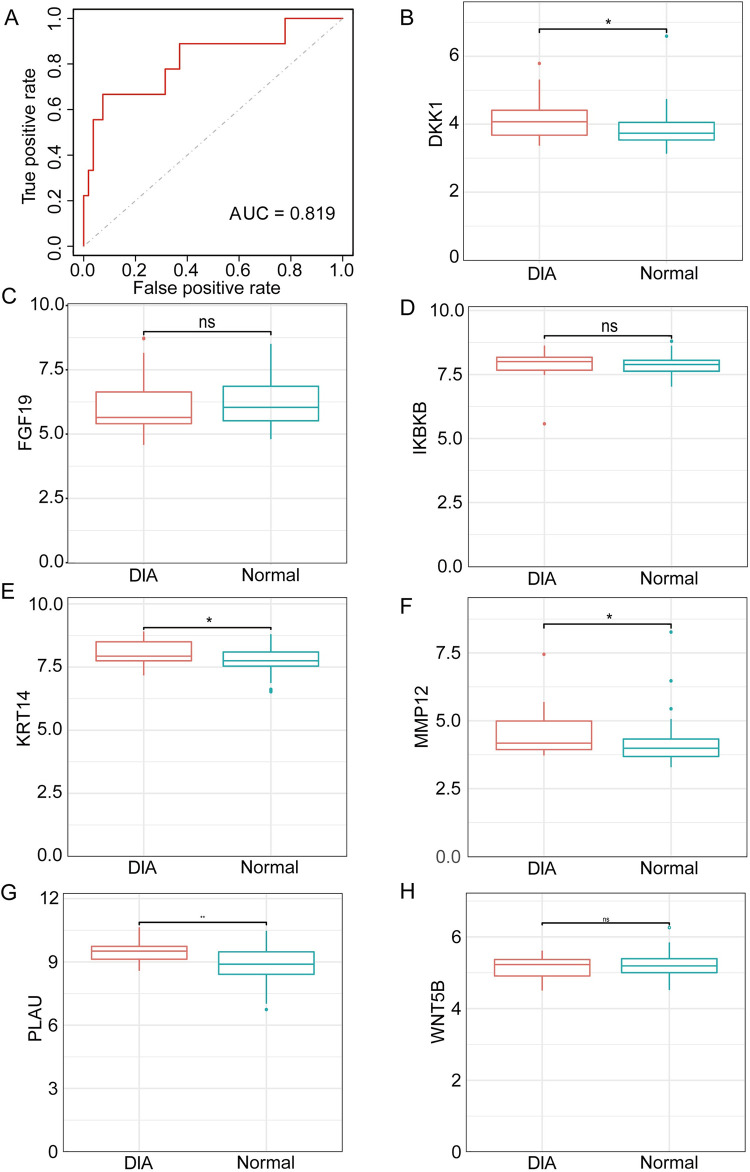
Validation of model genes for type 2 diabetes (T2D). (A) Receiver operating characteristic curves for T2D-related model genes from the GSE38642 dataset. (B–H) Boxplots of model gene expression in the validation dataset GSE41762.

LASSO analysis of the 11 hub genes using the minimum standard of one standard error (SE) generated seven model genes: *DKK1*, *MMP12*, *KRT14*, *PLAU*, *WNT5B*, *IKBKB*, and *FGF19* (Fig 4B and 4C). We then analyzed and visualized the relationships among these model genes (Fig 4D). Receiver operating characteristic (ROC) and AUC analysis was then conducted using the model genes, and the sensitivity and specificity of each was used for the diagnosis of ACP (Fig 5A). The expression of 7 model genes were evaluated in the validation dataset of ACP (Fig 5B–5H). Similarly, we perform ROC and AUC analysis on the T2D related dataset (Fig 6A). The expression of 7 model genes were then evaluated in the validation dataset of T2D (Fig 6B–6H). AUC values >0.8 were taken to indicate significant differential expression of the seven model genes for ACP and T2D, and a high diagnostic value for the differentiation of individuals with and without the disease. Boxplots showed that *DKK1*, *MMP12*, *KRT14*, and *PLAU* were significantly differentially expressed in both ACP and T2D, with higher expression in the tumor/disease group than in the non-tumor/non-disease group; and *WNT5B*, *IKBKB*, and *FGF19* exhibited significant differential expression in ACP, but not in T2D.

### Immune cell infiltration and its relationship with hub genes

**Fig 7:**
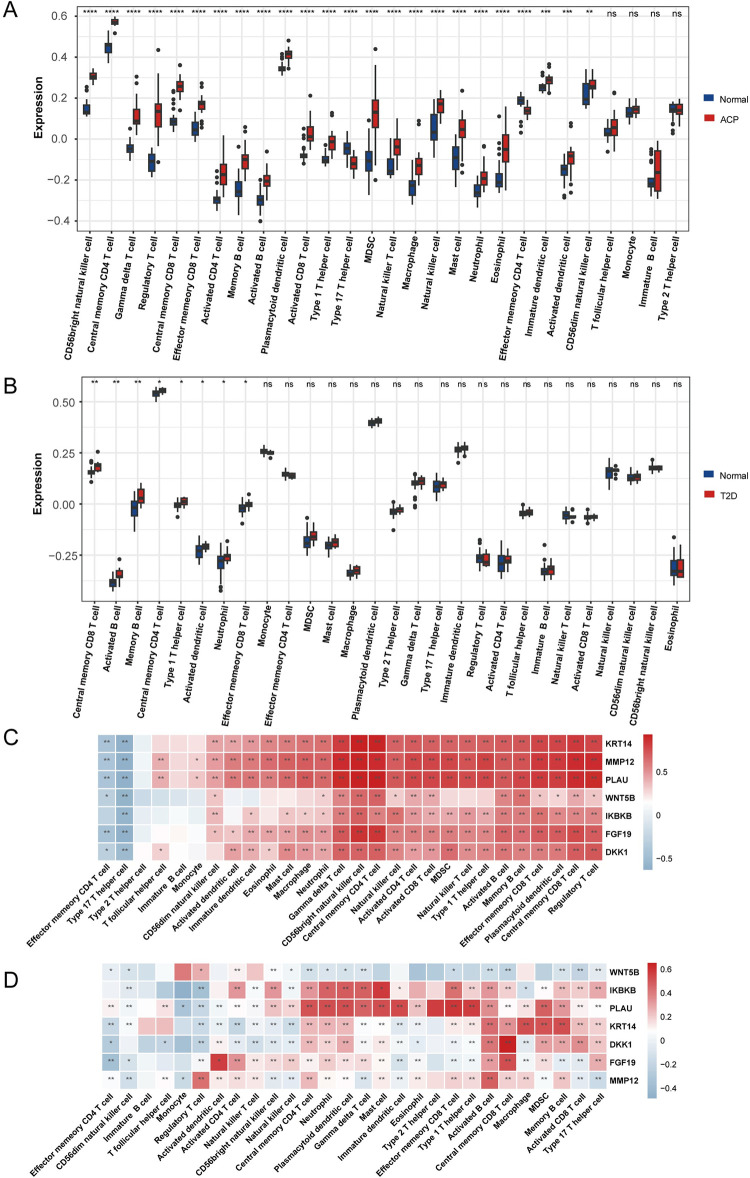
Analysis of the immune landscape associated with adamantinomatous craniopharyngioma (ACP) and type 2 diabetes (T2D). (A) Comparison of the infiltration of 28 types of immune cells in ACP tumors and non-tumor control tissue. (B) Comparison of the infiltration of 28 types of immune cell in the T2D disease and control groups. (C) Correlation coefficients for the relationships between seven model genes and the levels of immune cell infiltration in ACP. (D) Correlation coefficients for the relationships between the seven model genes and the levels of immune cell infiltration in T2D. * *p* < 0.05; ** *p* < 0.01; *** *p* < 0.001; **** *p* < 0.0001.

To characterize the differences in immune cell infiltration between the disease and control groups for patients with ACP and T2D, the ssGSEA algorithm was used to evaluate the distribution of 28 immune cell types in samples from the GSE94349 and GSE38642 datasets (Fig 7A and 7B). We identified greater infiltration with central memory CD8+ T cells, activated B cells, memory B cells, central memory CD4+ T cells, neutrophils, type 1 helper cells, activated dendritic cells, and effector memory CD8+ T cells in tissues affected by ACP and T2D than in healthy tissues, which implies that these cells may play roles in the progression of ACP and T2D. Furthermore, analysis of the associations between the 28 immune cell types and the hub genes showed that the hub genes were closely related to both diseases. For both diseases, we found positive correlations of the abundances of central memory CD8+ T cells, activated B cells, memory B cells, central memory CD4+ T cells, and neutrophils with *KRT14*, *MMP12*, *PLAU*, and *DKK1* expression (Fig 7C and 7D, S1–S4 Tables in S1 File). In addition, the abundances of central memory CD8+ T cells, activated B cells, memory B cells, central memory CD4+ T cells, and neutrophils negatively correlated with the expression of the *WNT5B* gene in patients with the two diseases, implying that it may play a role in immune cells in these diseases. These findings imply that immune cells play roles in the similar pathogenesis of ACP and T2D.

### Results of the functional enrichment analysis

**Fig 8:**
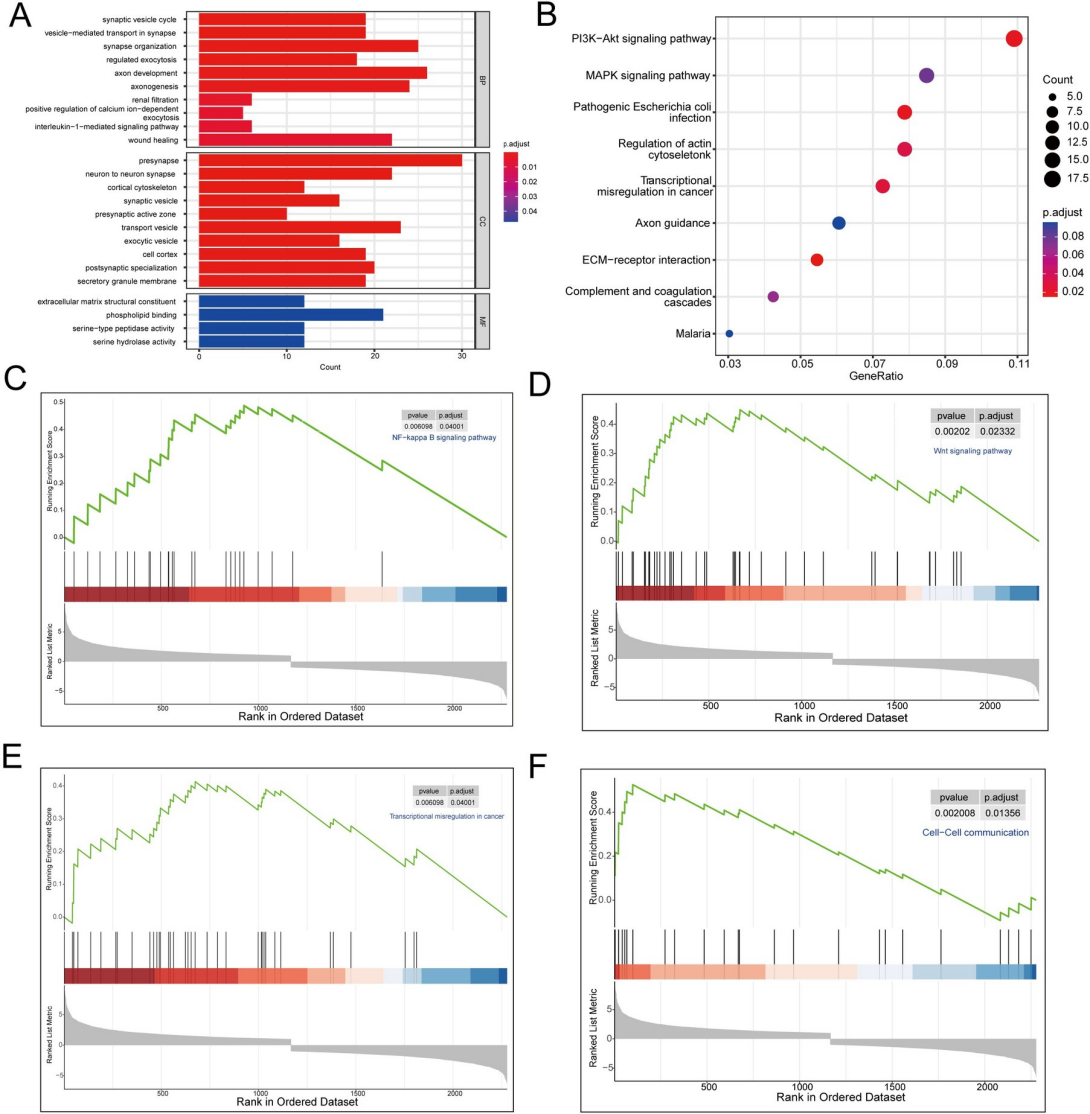
Functional enrichment analysis. (A) Bar graph of the top 10 enriched Gene Ontology terms derived from the differentially expressed genes (DEGs) in each category. BP, biological process; CC, cellular component; MF, molecular function. (B) Bubble plot of the Kyoto Encyclopedia of Genes and Genomes (KEGG) pathways that were significantly enriched by DEGs. (C–F) Upregulated pathways on Gene Set Enrichment Analysis (GSEA).

GO was used to analyze the biological functions of the DEGs. The top 10 enriched GO terms in the biological process (BP) category were found to be involved in interneuronal signaling, renal function, immune system regulation, and wound healing; and had biological functions related to synaptic vesicle release, intracellular substance transport, and neuron development. The top 10 enriched GO terms in the cellular component (CC) category were found to be related to the structure and functional regulation of neurons and cytoplasmic components in both groups. This included the formation and regulation of components such as synaptic vesicles, transport vesicles, secretion of substances, neuron precursors, neuronal synapses, cell cortex, and secretory granule membrane. Only four GO terms were enriched in the molecular function (MF) category: phospholipid binding, extracellular matrix structural constituent, serine-type peptidase activity, and serine hydrolase activity (Fig 8A). KEGG analysis revealed the enrichment of various pathways (Fig 8B), including the phosphoinositol 3-kinase-AKT signaling pathway, which has a crucial role in the regulation of cell proliferation, survival, and metabolism, and is associated with various diseases, including cancer, diabetes, and cardiovascular diseases. This pathway also regulates actin cytoskeleton remodeling, which is involved in the maintenance of cell morphology, cell movement, and signal transduction. The extracellular matrix-receptor interaction pathway is well known for its importance in the regulation of cell-environment interactions, cell adhesion, and signal transduction in biological processes [28]. In addition, GSEA of the DEGs showed significant upregulation of the Wnt signaling pathway, transcriptional dysregulation in cancer, the nuclear factor (NF)-kappa B signaling pathway, and cell–cell communication (Fig 8C–8F). These pathways and biological processes involve one or more hub genes that independently or collectively participate in important physiological processes and biological functions, such as cancer progression, cell development, and immune response.

## Discussion

In this study using a series of bioinformatics analyses, we found 11 hub genes commonly used in CP and T2D. Of these, seven (DKK1, MMP12, KRT14, PLAU, WNT5B, IKBKB, and FGF19) were also identified by least absolute shrinkage and selection operator analysis. Finally, MMP12, PLAU, KRT14, and DKK1 could be involved in the common pathogenetic mechanism of CP and T2D. In addition, we have characterized the differences in immune cell infiltration that characterize these two diseases. Hypothalamic obesity (HO) caused by CP and the hypothalamic damage induced by its treatment are one of the causes of severe atherosclerotic cardiovascular disease, T2D, and metabolic syndrome [29–31]. HO occurs in up to 50% of patients with CP, and up to 20% have obesity at the time of diagnosis [7, 32–34]. Pathological changes in the hypothalamus leads to the hypersecretion of glucocorticoids, which is a well-known risk factor for T2D [35–38]. In addition, the ventral nucleus of the hypothalamus plays a more important role in the regulation of glucose homeostasis [39–41]. Obesity causes local impairment of receptor-dependent insulin signaling across the blood-brain barrier and a consequent reduction in insulin sensitivity in the hypothalamus. This obesity-associated metabolic disorder ultimately leads to glucose intolerance and frank T2D [42–44]. However, the details of the pathogenesis of HO have not been fully elucidated. Excessive hunger caused by dysfunction of the satiety center may be an explanation, and damage to the ventral wall of the hypothalamus causes a reduction in vagal tone and the excessive secretion of insulin by pancreatic β cells, which in turn leads to HO [45–48]. However, a causal relationship between the greater insulin secretion and obesity has not been fully elucidated, and the pharmaceutical treatment of excessive insulin secretion does not control obesity [49–51]. T2D is often a consequence of having overweight or obesity, and most patients with T2D have one of these. However, the phenotype of obesity is not closely associated with the diabetic phenotype [52], and the prevalences of prediabetes and T2D are <50% in patients with obesity [53]. Damage to the hypothalamus caused by CP can lead to insulin resistance, which affects the glucose control of patients, and their preoperative insulin resistance is not related to their body mass [54]. Indeed, individuals with CP and the same body mass index as unaffected individuals have lower glucose tolerance and insulin sensitivity [55, 56].

Thus, HO does not seem to fully explain the similarities between CP and T2D. Therefore, we chose to characterize the molecular mechanisms through the analysis of similarly expressed genes. Using a comprehensive bioinformatic analysis, single-gene validation, and functional enrichment analysis, we have identified four highly associated genes: *DKK1*, *MMP12*, *KRT14*, and *PLAU*. Associations of *MMP12* with ACP or T2D have been previously reported, based on bioinformatic analysis and *in vitro* experiments, and these led to the suggestion that *MMP12* may be a therapeutic target for CP [17, 57]. It also plays an important role in the pathological changes associated with T2D and the related complications [58, 59].

The *DKK1* gene is involved in cancer transcriptional dysregulation, the Wnt signaling pathway, and other processes [60, 61]. The Wnt pathway and its components play important roles in metabolic disorders such as T2D [62, 63]. Over-activation of the Wnt signaling pathway is widely recognized as an important mechanism underlying ACP [64], as well as other cancers. The most well-known characteristic of the protein encoded by the *DKK1* gene is its role as an inhibitor of the canonical β-catenin-dependent Wnt pathway, in which it exerts its effects by blocking the binding of Wnt ligands to their co-receptors [65]. Typically, *DKK1* is expressed at low levels in most other normal human tissues; for example, in the heart, liver, lung, kidney, brain, and bone marrow [66]. Owing to its inhibitory effect on the β-catenin-dependent Wnt signaling pathway, DKK1 is considered to be a promising target for cancer therapy [67]. DKK1 is also known to have effects on bone metabolism, retinal pathology, and other parameters related to T2D [68, 69]. In the present study, we found that the expression of *DKK1* was high in patients with both diseases. However, the implications of this upregulation are not known. Therefore, further experimental study of its roles in both diseases and the mechanisms involved is required. Thus, DKK1 might serve as a diagnostic and/or therapeutic target for both diseases because of its dysregulation in both.

The findings of the GSEA in the present study indicate that the *PLAU* gene is connected with the NF-kB pathway. In T2D, NF-kB mediates chronic and subacute inflammation, leading to lower tissue insulin sensitivity [70]. Activation of the NF-kB pathway mediates inhibitory crosstalk at various levels of the insulin signaling pathway, which leads to hepatic insulin resistance [71]. In addition to reducing peripheral insulin sensitivity, the NF-kB pathway also affects glucose metabolism through its role in the central metabolic network of pancreatic islets. It promotes the destruction and dysfunction of pancreatic beta cells in response to metabolic stress and pro-inflammatory signals in patients with insulin resistance, which impairs compensatory insulin secretion, leading to impaired glucose tolerance and the development of T2D [72–75]. In cancer, activation of the classical NF-kB pathway promotes the proliferation, survival, angiogenesis, and invasion of tumor cells, contributing to tumor survival and progression [76, 77]. Anti-cancer drugs targeting various levels of the NF-kB pathway have been extensively used in clinical practice to counteract the pro-cancer effects of NF-kB pathway activation [78, 79]. In various cancer models, *PLAU* and NF-kB cooperate to promote cancer cell invasion [80]. However, there have been no studies of the joint action of NF-kB and *PLAU* in ACP. The results of the present analysis suggest that *PLAU* may mediate the shared pathogenic mechanisms involving the NF-kB pathway in ACP and T2D.

Intercellular communication mediated by hormones, growth factors, chemokines, cytokines, and neurotransmitters is crucial for processes such as morphogenesis, cell differentiation, homeostasis, cell growth, and cell–cell interactions [81–83]. In particular, intercellular communication in cancer and its implications for cancer therapy have been gaining attention [84, 85]. Of the present candidates, *KRT14*, a gene associated with intercellular communication, may play a crucial role in the development and progression of the diseases. Although an association between *KRT14* and tumor migration has been reported [86], there have been no studies of the relationships of KRT14 with T2D and ACP. This should be a focus for future research.

We also conducted immune cell infiltration analysis and a gene correlation analysis for the DEGs associated with both diseases. In our results, it was observed that there was a significant increase in infiltration by central memory CD8+ T cells, activated B cells, memory B cells, central memory CD4+ T cells, neutrophils, type 1 helper cells, activated dendritic cells, and effector memory CD8+ T cells compared to healthy tissues. This suggests that these cells may have significant roles in the progression of ACP and T2D. Moreover, analysis of the associations between 28 immune cell types and hub genes indicated a close relationship between these genes and both diseases. For both ACP and T2D, positive correlations were found between the abundances of central memory CD8+ T cells, activated B cells, memory B cells, central memory CD4+ T cells, and neutrophils with the expression of KRT14, MMP12, PLAU, and DKK1 genes. Additionally, these cell types showed negative correlations with WNT5B gene expression in patients with both diseases, suggesting a role in the immune response in these conditions. These findings seem to indicate similarities in the involvement of immune cells in the development of ACP and T2D. In other words, there seems to be a connection between the two diseases in immune-related physiological processes. It has been suggested that CPs are characterized by high levels of immunogenicity and infiltration by various immune cell types, and that this may be associated with the clinical outcomes [87–91]. Inflammation and the immune response play crucial roles in the pathogenesis of both the solid and cystic components of ACP [92]. Ayilavarapu *et al*. found an upregulation of the respiratory function of neutrophils in patients with T2D [93]. Although the role of neutrophils in metabolic dysfunction has not been analyzed, greater oxidative stress in the neutrophils of patients with diabetes may contribute to the oral complications of diabetes [94]. Immune cell-mediated inflammation and insulin resistance are involved in the pathogenesis of T2D. In addition, lymphocytes can promote local (adipose tissue) and systemic inflammation in patients with T2D through various mechanisms [95]. Therefore, immune cells might play important roles in the common aspects of the pathogenesis of these two diseases. Further experimental studies are needed to determine whether the highly correlated genes that we have identified affect disease pathogenesis through their effects on the immune system.

The present study had some limitations, such as its reliance on publicly available data, which may be subject to batch effects and involved a limited sample size. The analysis of immune cell infiltration may also be influenced by other biological factors, such as the tumor microenvironment and genetic mutations. Therefore, the present results require further validation in experimental and clinical studies.

In summary, using a comprehensive bioinformatics analysis, we have characterized the common features of ACP and T2D in terms of gene regulatory mechanisms, and identified four highly correlated genes. In addition, we have shown differential expression of *PLAU* and *DKK1* in ACP, and of *KRT14* in both diseases, and investigated their potential interactions in the pathogenesis of these diseases for the first time. We have also performed a cellular immune infiltration analysis, and have demonstrated relationships of these genes with the immune system. These findings reveal the similar expression of key genes in ACP and T2D, which improves our knowledge of the pathogenesis of ACP and T2D and the role of immunity. This study used bioinformatics analysis methods to focus on the common potential biomarkers for early detection and treatment of ACP and T2D for the first time. Helps bridge a critical gap in understanding the molecular connections between oncology and endocrinology, providing broader implications for studying the intersection of chronic disease and cancer. At the same time, to a certain extent, it provides new ways to deal with complex diseases by integrating multiple research methods such as molecular biology and immunology.

## Supporting information

S1 File (XLSX)
